# Deep-ultraviolet second-harmonic generation by combined degenerate four-wave mixing and surface nonlinearity polarization in photonic crystal fiber

**DOI:** 10.1038/s41598-017-10028-3

**Published:** 2017-08-23

**Authors:** Jinhui Yuan, Zhe Kang, Feng Li, Guiyao Zhou, Xianting Zhang, Chao Mei, Xinzhu Sang, Qiang Wu, Binbin Yan, Xian Zhou, Kangping Zhong, Kuiru Wang, Chongxiu Yu, Chao Lu, Hwa Yaw Tam, P. K. A. Wai

**Affiliations:** 1 State Key Laboratory of Information Photonics and Optical Communications (Beijing University of Posts and Telecommunications), P.O. Box 72 (BUPT), 100876 Beijing, China; 2 Photonics Research Centre, Department of Electronic and Information Engineering, The Hong Kong Polytechnic University, Hung Hom, Hong Kong; 3 The Hong Kong Polytechnic University Shenzhen Research Institute, Shenzhen, 518057 China; 4 Guangdong Provincial Key Laboratory of Nanophotonic Functional Materials and Devices, South China Normal University, 510006 Guangzhou, China; 5 Department of Physics and Electrical Engineering, Northumbria University, Newcastle upon Tyne, NE1 8ST United Kingdom; 6 Photonics Research Centre, Department of Electrical Engineering, The Hong Kong Polytechnic University, Hung Hom, Hong Kong

## Abstract

Deep-ultraviolet (UV) second-harmonics (SHs) have important applications in basic physics and applied sciences. However, it still remains challenging to generate deep-UV SHs especially in optical fibers. Here, for the first time, we experimentally demonstrate the deep-UV SH generations (SHGs) by combined degenerate four-wave mixing (FWM) and surface nonlinearity polarization in an in-house designed and fabricated air-silica photonic crystal fiber (PCF). When femtosecond pump pulses with average input power *P*_av_ of 650 mW and center wavelength *λ*_p_ of 810, 820, 830, and 840 nm are coupled into the normal dispersion region close to the zero-dispersion wavelength of the fundamental mode of the PCF, the anti-Stokes waves induced by degenerate FWM process are tunable from 669 to 612 nm. Then, they serve as the secondary pump, and deep-UV SHs are generated within the wavelength range of 334.5 to 306 nm as a result of surface nonlinearity polarization at the core-cladding interface of the PCF. The physical mechanism of the SHGs is confirmed by studying the dependences of the output power *P*_SH_ of the SHs on the PCF length and time. Finally, we also establish a theoretical model to analyze the SHGs.

## Introduction

Harmonic generations such as second-harmonic^1–4^, third-harmonic^5–7^, etc. are involved in different nonlinear processes, and can be used to generate new spectral components within ultraviolet to near-infrared wavelength region. Second-harmonic generation (SHG) as a second-order nonlinear optical phenomenon has been attracting much research interests because of important applications in biophysical imaging and multi-photon ionization^8–10^. Since the early 1980’s, SHGs in silica optical fibers^11–13^ and silicon-based waveguides^14–16^ have been reported. Compared with silicon-based waveguides, the SHG in optical fibers is limited in principle because silica glass as an amorphous material with inversion symmetry does not have second-order nonlinearity. Up to now, several methods, which include poling the optical fibers using the external light or electric fields^17–20^ and functionalizing the surface of the optical fiber tapers with different nonlinear materials^21–24^, were developed to induce second-order nonlinearity in the optical fibers. However, these methods suffer from thermo-instability and long-term instability of the induced second-order nonlinearity susceptibility, the difficulty in fabricating the ideal optical fiber tapers, and the necessity to consider the complex optical properties of the nonlinear materials.

Surface nonlinearity polarization at the core-cladding interface of the optical fibers is one of the dominant mechanisms of SHGs^25–27^, and does not have the challenges mentioned above. Since Terhune *et al*. theoretically investigated the formalism of such a nonlinear polarization which is induced by significant changes of the optical fiber structure and electric field near the interface region^25^, direct generations of second-harmonics (SHs) within the near-infrared to visible spectral regions from the incident pump based on surface nonlinearity polarization have been reported in optical fibers and photonic crystal fibers (PCFs)^28–30^. However, with only surface nonlinearity polarization, it is very difficult to directly generate deep-UV SHs from the pump pulses which are emitted by the existing mode-locked lasers centered at wavelengths of 1550, 1064, and 800 nm. In the past few years, cascaded nonlinear optical phenomena such as cascaded Raman and four-wave mixing (FWM) effects have been demonstrated to obtain visible and near-infrared wavelengths in the PCFs^31–34^. Based on cascaded FWM effect, it is possible that the new optical waves generated from the initial FWM process can serve as the secondary pump for the nonlinear process of the SHGs. Thus, the deep-UV SHs could be obtained by a combination of the FWM effect and surface nonlinearity polarization.

In this paper, we focus on studying the physical mechanism of the SHGs, and experimentally demonstrate for the first time deep-UV SHGs by combined degenerate FWM and surface nonlinearity polarization when femtosecond pump pulses are coupled into the normal dispersion region close to the zero-dispersion wavelength (ZDW) of the fundamental mode of an in-house designed and fabricated air-silica PCF. As the pump wavelengths change from 810 to 840 nm, the anti-Stokes waves, which are generated within the wavelength range of 669 to 612 nm from the initial FWM process, are used as the secondary pump. Then, based on surface nonlinearity polarization at the core-cladding interface of the PCF, the deep-UV SHs generated from the anti-Stokes waves are tunable from 334.5 to 306 nm.

## Results and Discussion

**Figure 1:**
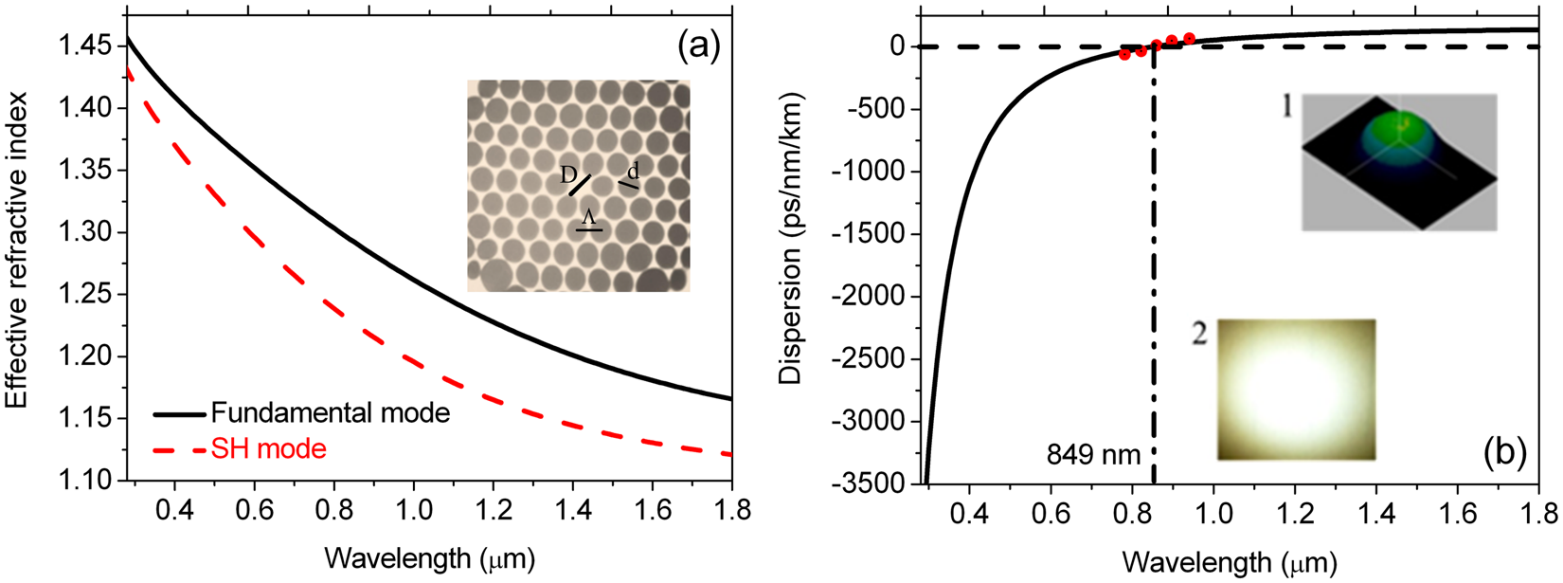
(**a**) The effective refractive index curves of the fundamental and SH modes calculated as functions of the wavelength. The inset shows the cross-sectional structure of the PCF used. (**b**) The calculated group-velocity dispersion profile of the fundamental mode with the zero-dispersion wavelength at 849 nm. The red solid dots correspond to the dispersion values measured by the pulse time-delay method. The insets 1 and 2 show the three-dimensional coupled-field profile observed at the input end of the PCF by a CCD camera and the output spatial far-field intensity distribution of the incident pump in the form of the fundamental mode, respectively.

We designed and fabricated the solid-core PCF from the purified silica material with the refractive index of ~1.46 by the stack and draw technique in our laboratory. The cross-sectional structure of the PCF is shown in the inset of Fig. 1(a), where the core diameter (*D*) and relative air-hole size (*d*/Λ) in the cladding region are 2.91 μm and 0.85, respectively. Because of the large *d*/Λ, three guided-modes can be supported in the fiber core, but we only focus on the characteristics of the fundamental and SH modes. Figure 1(a) shows the calculated effective refractive indices of the fundamental and SH modes considered. The group-velocity dispersion curve of the fundamental mode derived from the effective refractive index is shown in Fig. 1(b), which agrees well with the measured values by pulse time-delay method (the red solid dots). Thus, when femtosecond pulses at ~800 nm are used as the incident pump, the PCF is pumped in the normal dispersion region shorter than the ZDW of 849 nm. Insets 1 and 2 of Fig. 1(b) show the three-dimensional coupled-field profile observed at the input end of the PCF by a CCD camera and the output spatial far-field intensity distribution of the incident pump in the form of the fundamental mode, respectively. From insets 1 and 2 of Fig. 1(b), the incident pump is well coupled into the PCF and propagated in the fundamental mode. The corresponding nonlinear coefficient at ~800 nm calculated from the effective mode area is ~0.073 W^−1^m^−1^.

We used a commercial mode-locked Ti:sapphire laser (Mira 900) as the pump source, the operating wavelength of which is tunable from 780 to 900 nm and the pulse width is 120 fs. The pump pulses are coupled by a 40× microscope objective into the fundamental mode of a 30 cm long PCF by controlling the injection conditions (incident angle relative to the propagation axis, excitation radial position). The free-space coupling efficiency can be up to 65%. An optical isolator is employed to prevent the backscattered pump light into the laser cavity, and an adjustable attenuator is used to change the average input power. With the cut-back technique and a Xenon lamp as the light source, the propagation loss is measured to be above 1 dB/m around 300 nm. An optical spectrum analyzer (OSA) with a measurement range of 200 to 1100 nm is used to measure the optical spectra at the output end of the PCF. Several optical filters at different wavelengths inserted before the OSA are used to reduce the powers of the residual pump, anti-Stokes wave, and Stokes wave generated in order to prevent the OSA from damage.

**Figure 2:**
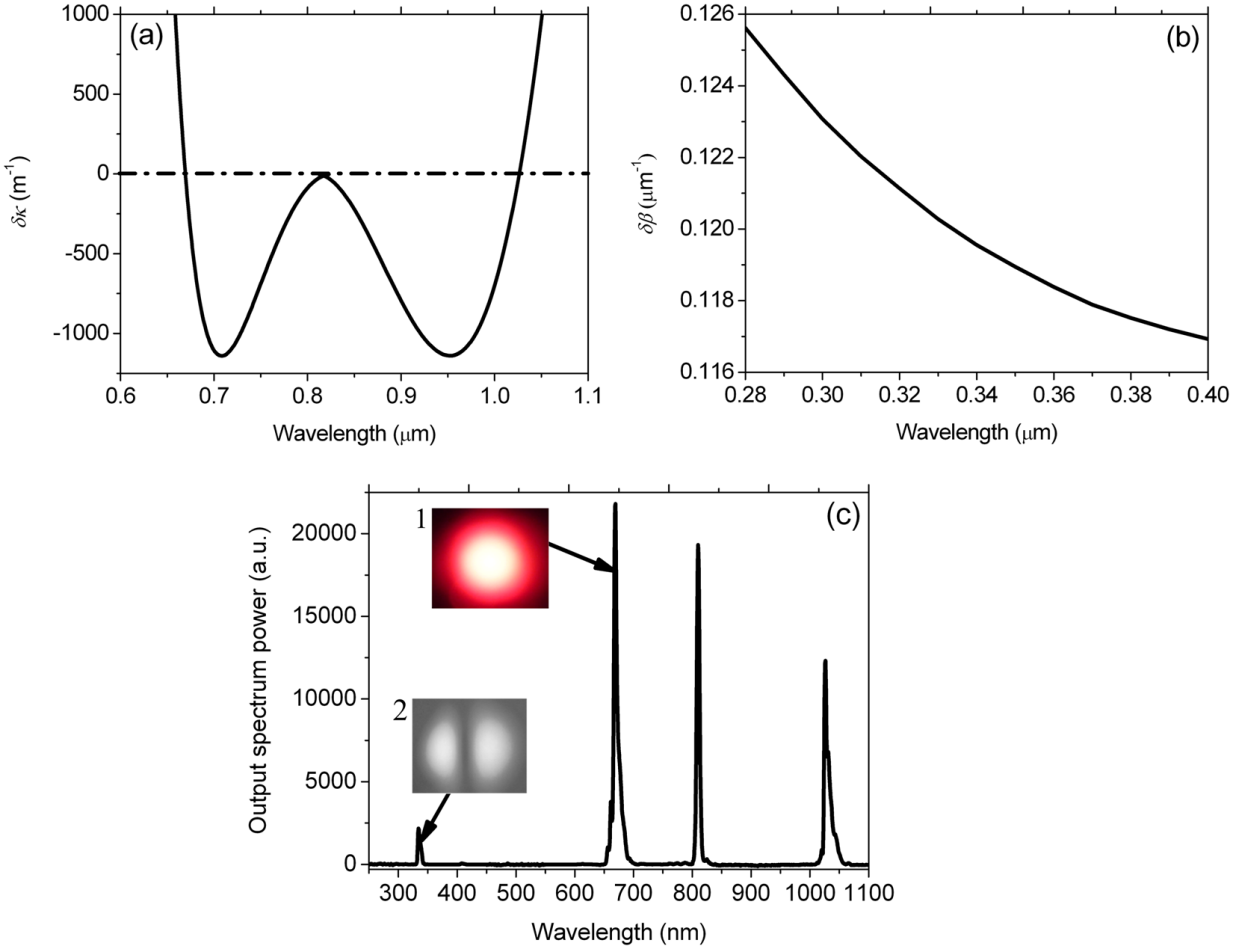
For femtosecond pump pulses with center wavelength *λ*_p_ of 810 nm and average input power *P*_av_ of 650 mW (the peak power of 78 kW), (**a**) The calculated phase-mismatch factor *δκ*, (**b**) the calculated phase-mismatch parameter *δβ* between the propagation constants *β*(*ω*_as_) and *β*(2*ω*_as_) of the anti-Stokes wave and SH, and (**c**) the output spectrum observed. The insets 1 and 2 in (**c**) show the output far-field distributions of the anti-Stokes wave and SH observed by the color and black-white-UV CCD cameras, respectively.

In the experiment, the center wavelength *λ*_p_ and average input power *P*_av_ of femtosecond pump pulses should be appropriately chosen to satisfy the phase-matching condition of the FWM process for efficient generation of the SHs. When femtosecond pump pulses with the center wavelength *λ*_p_ of 810 nm and average input power *P*_av_ of 650 mW (peak power of 78 kW) are launched into the fundamental mode of the PCF, the calculated *δκ* is shown in Fig. 2(a). It is noted that *δκ* reaches zero at the visible and near-infrared wavelengths of 668.9 and 1026.2 nm, respectively, along with the frequency shift amount from the initial pump of ~2601 cm^−1^. Figure 2(b) shows the calculated *δβ* between the propagation constants *β*(*ω*_as_) and *β*(2*ω*_as_) of the anti-Stokes wave and SH when the anti-Stokes wave generated is considered as the pump for the SHG. It can be seen from Fig. 2(b) that *δβ* gradually decreases within the considered wavelength range from 0.28 to 0.4 μm. Under the same pump condition, Fig. 2(c) shows the output spectrum observed from the output end of the PCF. From Fig. 2(c), because the center wavelength of femtosecond pump pulses is close to but shorter than the ZDW (849 nm) of the fundamental mode of the PCF, self-phase modulation (SPM) plays an important role in spectral broadening of the initial pump. As a considerable part of the pump energy is depleted, the anti-Stokes and Stokes waves are generated at the wavelengths of 669 and 1026.3 nm, respectively, which agree well with the theoretical results shown in Fig. 2(a). Moreover, the anti-Stokes wave generated at 669 nm serves as the secondary pump, and the discrete SH is observed at the deep-UV wavelength of 334.5 nm. The surface nonlinearity polarization, which is resulted from the local inhomogeneity at the core-cladding interface of the PCF, is considered as the dominant mechanism for the SHG. As seen from the insets 1 and 2 of Fig. 2(c), the far-fields of the anti-Stokes wave and SH, which are observed respectively by the color and black-white-UV CCD cameras at the output end of the PCF, show the spatial distribution of the fundamental and SH modes. In the nonlinear optical process, because no pump energy is spread into the anomalous dispersion region of the fundamental mode of the PCF, the related soliton dynamics leading to continuum generation will not occur. Thus, we obtain a clean output optical spectrum of the deep-UV SH. The normal dispersion and SPM will have a determining effect on the spectral and temporal waveforms of the anti-Stokes wave and SH generated. In addition, the SHs derived from the incident pump and Stokes wave are not observed. The main reasons are as follows. First, the pump energy is greatly depleted in the process of FWM-based frequency conversion. Second, compared with the anti-Stokes wave, the energy conversion from the pump to the Stokes wave is lower. Third, the diffraction loss of the Stokes wave located at the longer wavelength which is propagated in such a small fiber core is large.

**Figure 3:**
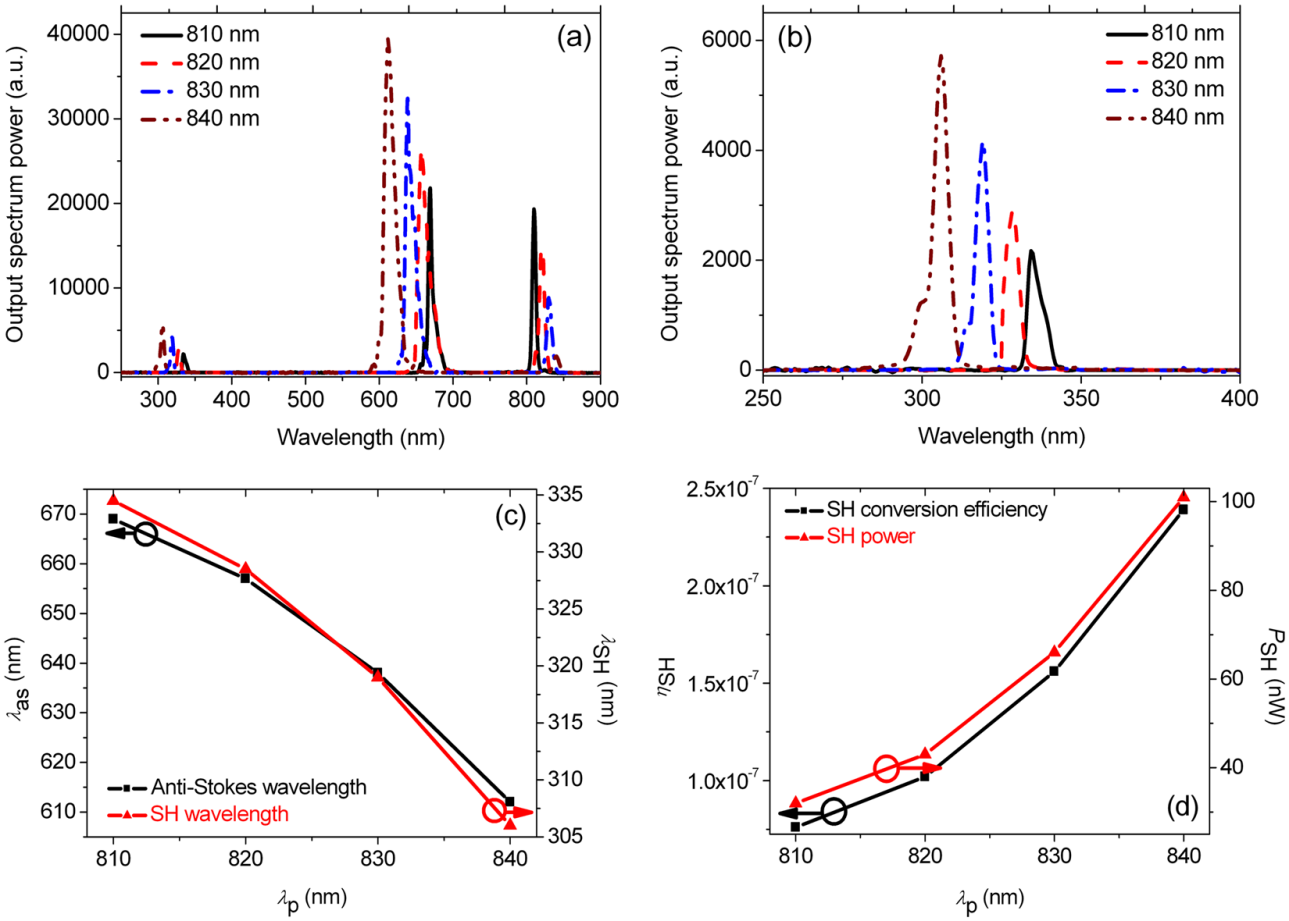
(**a**) The observed output spectra when femtosecond pump pulses with center wavelength *λ*_p_ of 810, 820, 830, and 840 nm and average input power *P*_av_ of 650 mW (the peak power of 78 kW) are used, (**b**) the zoom-in spectra of the SHs generated, (**c**) the wavelengths *λ*_as_ and *λ*_SH_ of the generated anti-Stokes waves and SHs as functions of *λ*_p_, and (**d**) the measured output power *P*_SH_ and corresponding conversion efficiency *η*_SH_ of the SHs as functions of *λ*_p_.

In Figs. 3(a,b), the observed full output spectra and zoom-in spectra of the SHs are shown when *λ*_p_ of femtosecond pump pulses at *P*_av_ of 650 mW is changed from 810, to 820, to 830, and to 840 nm, respectively. It can be seen from Figs. 3(a,b) that as *λ*_p_ gradually approaches the ZDW of the fundamental mode of the PCF, more pump energy is converted into the anti-Stokes waves centered at visible wavelengths of 669, 657, 638, and 612 nm and the Stokes waves located at longer near-infrared wavelengths which are beyond consideration here, and the corresponding SHs generated at the deep-UV wavelengths of 334.5, 328.5, 319, and 306 nm are evidently enhanced. The phase-matching between the fundamental and SH modes, which depends on the dispersion characteristic of the PCF used and the combined effect of the degenerate FWM and surface nonlinearity polarization, can be achieved within a wavelength range of 28.5 nm. Compared with the gas-filled hollow-core PCF^7^, it is possible to generate the SHs within a broader wavelength range in such a solid-core PCF. Figure 3(c) shows the relationships between the wavelengths *λ*_as_ and *λ*_SH_ of the anti-Stokes waves and SHs and *λ*_p_. The approximately linear variations with larger slopes indicate that *λ*_as_ and *λ*_SH_ can be widely tunable as *λ*_p_ changes. Estimated by Manley-Rowe relations of photon conservation, more than 40% of the total output power is contained in the anti-Stokes wave at wavelength of 612 nm. An optical filter with optical density of 0.06 and a nano-watt powermeter with sensitivity of 100 pW are combined to measure the output power of the SHs. The optical filters and nano-watt powermeter are combined to measure the output power of the SHs. Figure 3(d) shows the dependences of the measured output power *P*_SH_ of the SHs and the conversion efficiency *η*_SH_ from the incident pump to the SHs on *λ*_p_. For the coupling efficiency of 65% achieved, the pump power at the incident end of the PCF is up to 422.5 mW. As *λ*_p_ is changed from 810, to 820, to 830, and to 840 nm, *P*_SH_ are measured to be 32, 43, 66, and 101 nW, and the corresponding *η*_SH_ increases from 0.76 × 10^−7^, to 1.02 × 10^−7^, to 1.56 × 10^−7^, and to 2.39 × 10^−7^, respectively.

**Figure 4:**
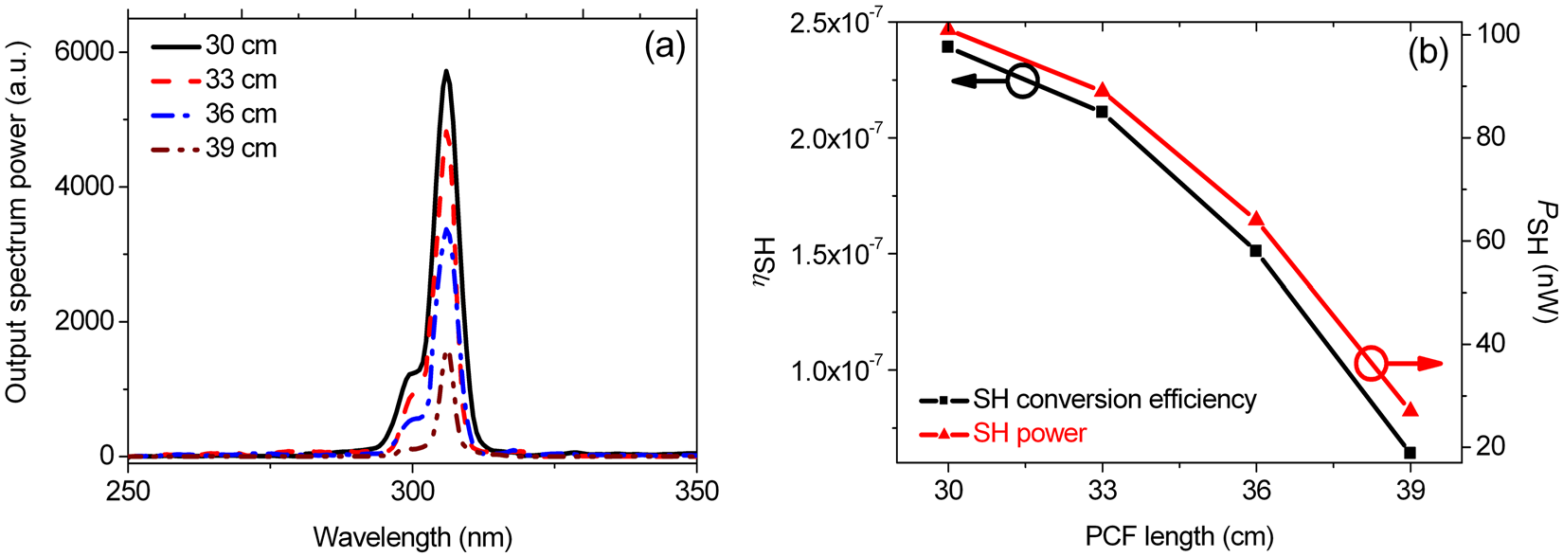
For femtosecond pump pulses with center wavelength *λ*_p_ = 840 nm and average input power *P*_av_ = 650 mW (peak power equals 78 kW), when the PCF length is changed from 30, to 33, to 36, and to 39 cm, respectively, (**a**) the observed output spectra of the SHs, and (**b**) the measured output power *P*_SH_ and conversion efficiency *η*_SH_ of the SHs as a function of the PCF length.

**Figure 5:**
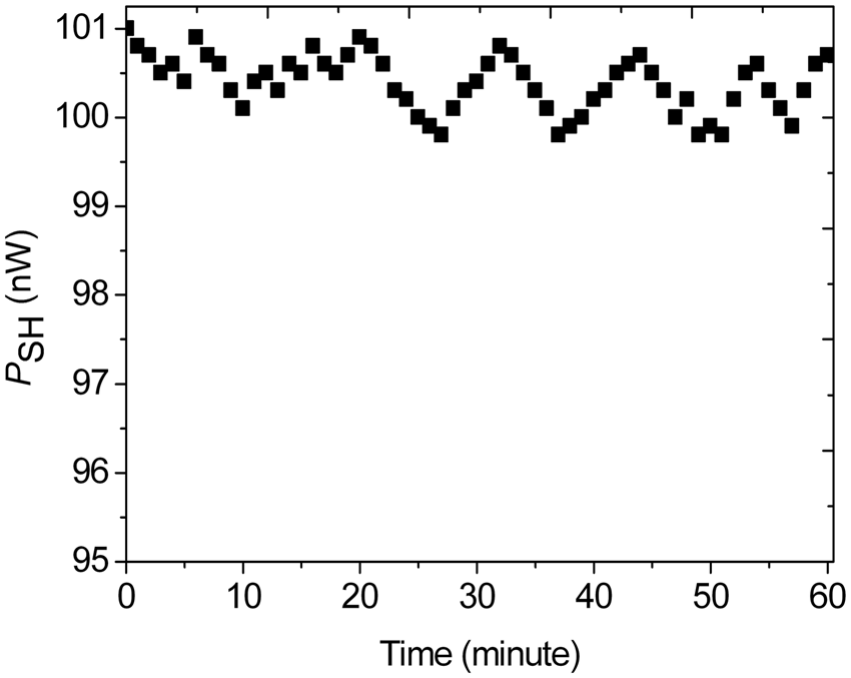
Dependence of the measured output power *P*_SH_ of the SHs from the 30 cm long PCF on the time for femtosecond pump pulses with center wavelength *λ*_p_ = 840 nm and average input power *P*_av_ = 650 mW (the peak power of 78 kW).

In the following, we will show that the physical mechanism of the SHGs in the experiment is related to the characteristics of the PCF used, namely surface nonlinearity polarization, not the bulk coupling optics of the silica material and dipole formation of the silica glass. For femtosecond pump pulses with *λ*_p_ = 840 nm and *P*_av_ = 650 mW, Figs. 4(a,b) show the observed output spectra of the SHs and the measured variations of *η*_SH_ and *P*_SH_ when the PCF length is changed from 30, to 33, to 36, and to 39 cm, respectively. As shown in Figs. 4(a,b), *η*_SH_ and *P*_SH_ of the SHs generated decrease as the length of the PCF increases. It can be seen from Fig. 4(b) that *P*_SH_ is reduced from 101, to 87, to 61, and to 25 nW, and the corresponding *η*_SH_ decreases from 2.39 × 10^−7^, to 2.06 × 10^−7^, to 1.44 × 10^−7^, and to 0.59 × 10^−7^, respectively. The increased propagation loss around 300 nm in longer PCFs is considered as the main reason for the decreases in *P*_SH_ and *η*_SH_. In addition, we also study the temporal dependence of *P*_SH_ under the same pump condition, as shown in Fig. 5. From Fig. 5, when femtosecond pump pulses are propagated inside a span of 30 cm long PCF, *P*_SH_ reaches the maximum value of 101 nW in only a few seconds, and remains relatively stable over ~60 minutes. The experimental results and observations adequately indicate that the SHGs in our work originate from the second-order nonlinearity which is induced by the surface nonlinearity polarization at the core-cladding interface of the PCF, not contributed from the bulk silica material or the dipole formation of the silica glass structure caused by the pump light field, which requires buildup time of tens of minutes to a few hours at the beginning stage of the SHGs and gradually decays over the time^35, 36^.

Finally, we will establish a theoretical model to analyze the SHGs based on the surface nonlinearity polarization and calculate the conversion efficiency^25, 37, 38^. For an *m*-th order guided-mode propagating along the PCF, the electric field *E*_m_(*ω*) and magnetic field *H*_m_(*ω*) can be described as

1 $${E}_{m}(\omega )={a}_{m}(\omega ,z){e}_{m}(\omega ,x,y)\,\exp \{i[{\beta }_{m}(\omega )z-\omega t]\},$$

2 $${H}_{m}(\omega )={a}_{m}(\omega ,z){h}_{m}(\omega ,x,y)\,\exp \{i[{\beta }_{m}(\omega )z-\omega t]\},$$

where *a*_m_(*ω*, *z*) is the scalar mode amplitude, which is a function of the angular frequency *ω* and propagation distance *z, e*_m_(*ω*, *x*, *y*), *h*_m_(*ω*, *x*, *y*) represent the mode field distributions across the cross-section (*x*, *y*) of the PCF’s core region, and *β*_m_(*ω*) is the propagation constant of the mode along the positive *z* direction.

The modes propagating in the PCF are described by using the HE representation. We assume that *a*_m_(*ω*, *z*) is a slowly varying function of *z*, and consider the frequency-doubling of the pump wave propagating as the fundamental mode (*m* = 1) at *ω* to create a SH mode (*m* = 2) at 2*ω*. Within the nonlinear coupling length *l*_NL_, near index-matching condition between the fundamental and SH modes as a function of *a*_1_, *a*_2_, *z*, *l*_NL_, and nonlinear polarization *P*_NL_ can be achieved.

We exclude the bulk effect of silica material, and only consider *P*_NL_ at the core-cladding interface of the PCF. The electric fields vary rapidly in the interface region because of the discontinuous effective refractive index. The gradients of the electric field in the core-cladding interface can lead to the SHG through *P*_NL_, which is dependent of the electric field components on the core side of the core-cladding interface. When the cylindrical coordinate system is used, *P*_NL_ inside the interface region can be described as

3 $$[\begin{array}{c}{P}_{r}(2\omega )/{\varepsilon }_{0}\\ {P}_{\varphi }(2\omega )/{\varepsilon }_{0}\\ {P}_{z}(2\omega )/{\varepsilon }_{0}\end{array}]=[\begin{array}{ccc}{E}_{r}^{2}(\omega ) & 0 & {E}_{r}^{2}(\omega )+{E}_{\varphi }^{2}(\omega )+{E}_{z}^{2}(\omega )\\ 0 & {E}_{r}(\omega )\,{E}_{\varphi }(\omega ) & 0\\ 0 & {E}_{r}(\omega )\,{E}_{z}(\omega ) & 0\end{array}]\,[\begin{array}{c}L\\ M\\ N\end{array}],$$

where the parameters *L*, *M*, and *N* can be calculated by considering the interface contribution within *l*_NL_^25^, and *ε*_0_ = 8. 854187817 × 10^−12^ F/m is the vacuum dielectric constant.

In the nonlinear optical process of SHG, the conversion efficiency of SH can be calculated by combining the coupled nonlinear equations in ref. 25 and Eqs. (1)–(3). For femtosecond pump pulses with *λ*_p_ = 840 nm and *P*_av_ = 650 mW (peak power = 78 kW) and the PCF length of 30 cm, the calculated *η*_SH_ is on the order of 10^−6^ when the fiber length-dependent phase-mismatch and fiber core non-uniformity along the *z* direction are considered^25^. The theoretical calculation supports the conjecture that the physical mechanism of the SHGs in our work is dominated by surface nonlinearity polarization. The difference between the theoretical and experimental (2.39 × 10^−7^) results could be due to propagation loss including the silica material absorption, core-cladding structure scattering, and leakage losses. Moreover, the longitudinal variation of the PCF geometric structure would result in the fluctuation of the dispersion profile of the fundamental mode, which will have detrimental effect on the FWM process and the efficiency of the SHGs. Methods to enhance the conversion efficiency and output power of the SHs include purifying the silica material to reduce its UV absorption loss, optimizing the fiber structure parameters, and improving the fabrication technique to reduce the scattering and leakage losses such that good fiber longitudinal uniformity is obtained. In addition, we can also choose the appropriate fiber length to reduce the propagation loss and employ high power pump laser to increase the output powers of the anti-Stokes waves generated as the secondary pump.

## Conclusion

In summary, deep-UV SHs within the wavelength range of 334.5 to 306 nm are experimentally demonstrated for the first time when femtosecond pump pulses are coupled into the normal dispersion region close to the ZDW of the fundamental mode in an in-house designed and fabricated air-silica PCF. The SHGs are realized by the combined degenerate FWM and surface nonlinearity polarization at the core-cladding interface of the PCF. Our findings from the study of the fundamental physical mechanism of SHGs in PCFs provide a way to obtain deep-UV SHs, which would have important applications in basic physics and applied sciences, such as surfaces and interfaces in physical system^39^, multi-photon ionization^40^, etc.

## Methods

### Phase-matching calculation for the FWM process

The degenerate FWM as a third-order parametric process can efficiently transfer energy from the pump wave to the two new optical waves when the matching conditions of the frequencies and the wave-vectors are satisfied at the same time. In the process of energy conversion, two incident pump photons at initial frequency *ω*_p_ are annihilated, while an anti-Stokes photon and a Stokes photon at up- and down-shifted frequencies *ω*_as_ and *ω*_s_ are simultaneously created, that is, 2*ω*_p_ = *ω*_as_ + *ω*_s_. Moreover, the phase-mismatch factor *δκ* induced by the silica material, fiber structure, and nonlinearity can be written as *δκ* = 2*β* (*ω*_p_) − *β* (*ω*_as_) − *β(ω*_s_) − 2*γP*, where *β*(*ω*_p_), *β*(*ω*_as_), and *β*(*ω*_s_) are the wave-vectors of the pump, anti-Stokes wave, and Stokes wave, respectively, *γ* is the wavelength-dependent nonlinear coefficient, and *P* is the pump peak power. Significant FWM process can occur only if *δκ* = 0 is satisfied.

### Phase-matching calculation for the SHG process

For the SHG process, two pump photons at frequency *ω*_p_ are up-converted to one SH photon with twice the incident pump optical frequency of *ω*_p_. The phase-mismatch parameter *δβ* between the fundamental and SH modes can be described as *δβ* = *β*(2*ω*_p_) −2*β*(*ω*_p_), where *β*(*ω*_p_) and *β*(2*ω*_p_) represent the propagation constants of the fundamental pump beam and its SH, respectively.
